# College and Hospital Notes

**Published:** 1899-11

**Authors:** 


### COLLEGE AND HOSPITAL NOTES.

A NEW hospital is building at Richmond, Va, which will, when completed, cost considerably more than $100,000. It will be admirably located at the corner of Twelfth and Broad Streets, where ample ground has been secured. The work of removing the buildings at present located thereon will soon begin. In a reasonable time the structure, which is to shelter the noble charity, will have been erected and its doors thrown open for the reception of patients.

The Richmond *Dispatch*, of September 28, 1899, in giving a detailed description of the site and the plans of the projectors says:

The corporators of the company are Messrs. R. S. Bosher, John L. Williams, E. T. D. Myers, William R. Trigg, John Skelton Williams, E. Randolph Williams, E. L. Bemiss, W. M. Habliston, C. W. Tanner, Fred Nolting, Dr. George Ben Johnston, and Dr. Christopher Tompkins. It would be difficult to obtain Richmond men of surer standing in the world of business, or in the professional world.

The institution will be of size sufficient to accommodate 150 patients. This includes the charity wards as well as the private rooms. Of course, like all such institutions, the hospital will be open both to patients who are able to pay for a private room and special attendants, as well as to the poor devil who hardly has where to lay his head when well and no rainy-day sum to pay the cost of a long illness.

The hospital will be under the control of a board of trustees. Medical and surgical service will be rendered by the staff of the Medical College of Virginia exclusively, except, of course, when other physicians and surgeons are especially invited to lend their assistance.

The establishment of this hospital marks the inauguration of the greatest charity Richmond has ever known. At present the Almshouse is the only city hospital in existence. The need of an ample modern hospital, where the unfortunate poor of the city may be treated has been felt for many years. The city has not been able to establish a charity hospital. The philanthropy of her citizens will do that which the municipality could not do.

Dr. George Ben Johnston, one of the corporators, and among the best known surgeons of America, stated last night that the hospital would be as thoroughly equipped as any in existence. Every scientific appliance known to medicine and surgery will be at the hand of the men who will have the hospital in charge. In this connection it should be stated that Dr. George Ben Johnston and Dr. Ennion G. Williams have been chiefly instrumental in inaugurating the movement for the establishment of the hospital.

Dr. Christopher Tompkins, who is the other physician among the corporators, a professor in the Medical College of Virginia, and one of the best known doctors in the city, stated to a *Dispatch* writer last night that the senior member of the well-known firm of architects of this city, Messrs. Nolting & Baskerville, had been in New York and Philadelphia some time this summer studying the forms of hospital architecture to be found there, with a view to applying his ideas in the plans for the great Richmond institution. However, an architect has not been engaged, and plans have not been adopted.

It is hardly possible to exaggerate the importance of the establishment of this great charitable enterprise in Richmond. Its establishment seems the climax of an era of unprecedented prosperity in the history of the city. Heretofore development along industrial lines has chiefly characterised the ushering in of the new era. It is a cause for sincere congratulation that charity is not to be forgotten by those who are making Richmond take such a high place in the world of business.

---

A VACANCY having occurred in the superintendency of the Utica State Hospital by the resignation of Dr. G. Alder Blumer, a civil service examination has been held to determine the qualifications of his successor. As a result the following-named candidates have been declared eligible, ranking in the order named: Harold Palmer, State Hospital, Utica; Henry P. Frost, State Hospital, Buffalo; William L. Russell, State Hospital, Willard; Jelvin Courtney, Hudson River State Hospital, Poughkeepsie. The first named has been appointed.

---

THE department of anatomy at the University of Buffalo has been enlarged considerably of late. The staff of teachers as now constituted is as follows: John Parmenter, professor of anatomy and clinical surgery; William C. Phelps, professor of surgical anatomy; Abram T. Kerr, adjunct professor and demonstrator of anatomy; James A. Gibson, assistant demonstrator of anatomy; James E. King, N. C. Russell, William House, Norman L. Burnham, Laurence Hendee, Amos T. Baker, Marshall Clinton, Herman K. DeGroat and Jacob Otto, assistants in anatomy.

---

A HOSPITAL at Batavia has long been talked of and still longer needed. Lately, however, there seems to be some prospects of crystalisation of the plans. Dr. B. F. Showerman has taken hold of the matter in earnest and has great hopes of success. He recently outlined his general plan to a reporter of the *Buffalo Evening News*. Among other things he said :

The management of the hospital is to be in the hands of a board of managers composed of doctors in this village. Every physician, who has a patient who has purchased a ticket entitling him to admission to the hospital, will have the right to take him there and treat him, either prescribing or furnishing the medicines needed, and will charge for his professional services just the same as if the patient was in his own home.

In cases of accidents, or if the person is unable to pay, let the village pay for his care, or let the churches or some society or benevolent person subscribe money to endow cots for such emergencies.

No one must expect that he can buy a ticket and be admitted to the hospital after he is sick. He must purchase his ticket when they are issued or before he becomes ill. The object of this enterprise is to insure a person a place to go and be care for during sickness, and not to give them that privilege after they have become sick.

The cost of equipping such a hospital, as I have described, and running it for one year would be about $6,000 or $7,000. It would probably cost nearly that amount the second year, but after that I think it could be run for $5,000 a year. I know of no reason why one or two thousand tickets should not be sold at once.

The well-know enterprise of Batavia ought to be a guarantee of the success of this plan. The necessities for such an institution are great, and the project as outlined is just and comparatively inexpensive.

---

THE governors of the New York Skin and Cancer Hospital, Second Avenue, corner 19th Street, announce that Dr. L. Duncan Bulkley will give a second series of clinical lectures on diseases of the skin in the outpatient hall of the hospital, on Wednesday afternoons, commencing November 1, 1899, at 4.15 o'clock. The course will be free to the medical profession.

For several years past Dr. Bulkley has endeavored to utilise the clinical material at the dispensaries for the benefit of the general profession, and last year a series of lectures were given that were well attended. Every physician interested in the study of this special branch of medicine will find these lectures most helpful.

